# Irisin as a Novel Biomarker of Subclinical Atherosclerosis in Severe Obesity

**DOI:** 10.3390/ijms24098171

**Published:** 2023-05-03

**Authors:** Júlia Carmona-Maurici, Araceli Rosa, Natalia Azcona-Granada, Elionora Peña, David Ricart-Jané, Anna Viñas, Maria Dolores López-Tejero, Joan Carles Domingo, Antonio Miñarro, Juan Antonio Baena-Fustegueras, Julia Peinado-Onsurbe, Eva Pardina

**Affiliations:** 1 Departament de Bioquímica i Biomedicina Molecular, Facultat de Biologia, Universitat de Barcelona, Diagonal 643, 08028 Barcelona, Spain; 2 Secció de Zoologia i Antropologia Biològica, Departament de Biologia Evolutiva Ecologia i Ciències Ambientals Facultat de Biologia, Universitat de Barcelona, 08028 Barcelona, Spain; 3 Institut de Biomedicina de la Universitat de Barcelona (IBUB), 08028 Barcelona, Spain; 4 Centre for Biomedical Research Network on Mental Health (CIBERSAM), Instituto de Salud Carlos III, 28029 Barcelona, Spain; 5 Departament de Genètica, Microbiologia i Estadística, Facultat de Biologia, Universitat de Barcelona, 08028 Barcelona, Spain; 6 Gastrointestinal Surgery Department, Arnau de Vilanova University Hospital, IRB Lleida, University of Lleida, 25198 Lleida, Spain

**Keywords:** irisin, severe obesity, metabolic disorders, subclinical atherosclerosis, bariatric surgery, *FNDC5* gene, rs3480, genetic variability

## Abstract

Severe obesity (SO) can accelerate atherosclerosis and the onset of acute cardiovascular events. The diagnosis of atherosclerosis in the context of a high body mass index (BMI) can be challenging, making the identification of biomarkers clinically relevant. We aimed to assess the usefulness of irisin as a biomarker for subclinical atherosclerosis in participants with SO. This prospective observational study included 61 participants undergoing bariatric surgery for SO, defined as a BMI >40 kg/m^2^ or >35 kg/m^2^ with at least one comorbidity. Atherosclerotic plaques were detected by ultrasound. Plasma samples were obtained 1 month before and at 6 and 12 months after bariatric surgery to measure irisin by ELISA. Additionally, subcutaneous samples of adipose tissue were taken and genotyped to identify irisin polymorphism rs3480. Irisin levels were positively correlated with BMI (*r* = 0.23, *p* = 0.0064), negatively correlated with atheroma-related parameters (e.g., carotid intima-media thickness), and lower in subjects with atheroma (*p *< 0.0002). Irisin also showed good overall accuracy for discriminating plaque presence (AUC, 0.81; 95% CI, 0.6956–0.9156). However, the rs3480 polymorphism correlated with neither the irisin levels nor the presence of atheromas. Iirisin could identify subclinical atherosclerosis in SO and might facilitate clinical diagnosis.

## 1. Introduction

The prevalence of overweight and obesity has increased globally over recent decades to become a major public health challenge. Currently, 13% of adults in the world have obesity and 8% of global deaths were attributed to obesity [1]. Severe obesity (SO) is a complex chronic disease that can lead to serious health conditions [2]. Some of these comorbidities can revert after bariatric surgery (BS), which is currently the most effective approach for treating SO [3]. Moreover, although SO is a well-known risk factor for atherosclerosis, the mechanisms underlying this association are not fully understood [4].

Atherosclerosis is a chronic disease that begins with the formation of atheromas and predisposes individuals to cardiovascular disease (CVD) and increased mortality [5]. An estimated 17.9 million people died from CVDs in 2019, representing 32% of all global deaths [6]. Carotid ultrasound is the recommended method to detect subclinical atheromatous plaques [7], but the fat accumulation in cases of SO can make the diagnoses based on arterial wall imaging challenging [8]. Accordingly, the identification of the biomarkers of subclinical atherosclerosis is clinically relevant in the context of SO. Our previous research in this area has revealed abnormalities in several molecules implicated in the metabolic and inflammatory mechanisms that also promote atherogenesis [9]. Adipose and muscle tissues play key roles in producing many of these molecules, among which the novel adipomyokine irisin appears to promote metabolic health and affect CVD [10,11]. Hence, it seems reasonable to suggest that irisin could be involved in both obesity and atherosclerosis.

Concerning energy metabolism, irisin induces the upregulation of uncoupling protein-1 and the conversion of white adipose tissue to brown adipose tissue, resulting in heightened thermogenesis and energy expenditure [12]. In this way, conflicting results suggest that irisin may play a direct role in obesity [13,14]. Current knowledge of the biological functions of irisin also includes regulating glucose and lipid metabolism, anti-inflammation, and anti-oxidation [15,16,17,18]. The dysregulation of these processes impairs metabolic responses, which in turn, can lead to atherosclerosis [9,19]. Nevertheless, the role of irisin in the development of atherosclerosis requires further research to understand its mechanisms of action fully [10,11]. To the best of our knowledge, no previous studies in the context of SO and BS have considered the use of irisin as a serological and genetic biomarker (*FNDC5* gene) for detecting subclinical atherosclerosis. Accordingly, research to elucidate the functions of irisin might provide new opportunities for developing more effective approaches for early diagnoses of atherosclerosis and preventing CVD.

This study aimed to assess the usefulness of irisin as a biomarker for subclinical atherosclerosis in cases of SO. To accomplish our main objective, we propose the following specific aims: (i) to analyze the serum levels of irisin and its genetic variability in cases with and without atheroma undergoing BS, (ii) to evaluate the association between irisin levels and the biochemical parameters involved in atherosclerosis, and (iii) to assess the usefulness of irisin as a biomarker of subclinical atherosclerosis in patients with SO undergoing BS.

## 2. Results

### 2.1. General Characteristics

Table 1 summarizes the anthropometric and biochemical characteristics of the study groups with and without atheromatous plaques. Atheromatous plaques did not remit 12 months after BS, but the BMIs decreased by about 33% after BS, and did not differ statistically between groups. Regarding biochemical parameters, triacylglycerides (TAG), glucose, glycated hemoglobin (Hba1c), and Homeostatic Model Assessment for Insulin Resistance (HOMA-IR) results were significantly higher in subjects with plaques, but they did decrease after BS. Referring comorbidities, SO and atheroma were also associated with a higher incidence of hypertension, dyslipidemia, and type 2 diabetes mellitus (T2DM), but their prevalence also decreased after BS.

### 2.2. Irisin Levels and Genetic Variability in Subclinical Atherosclerosis

**Figure 1:**
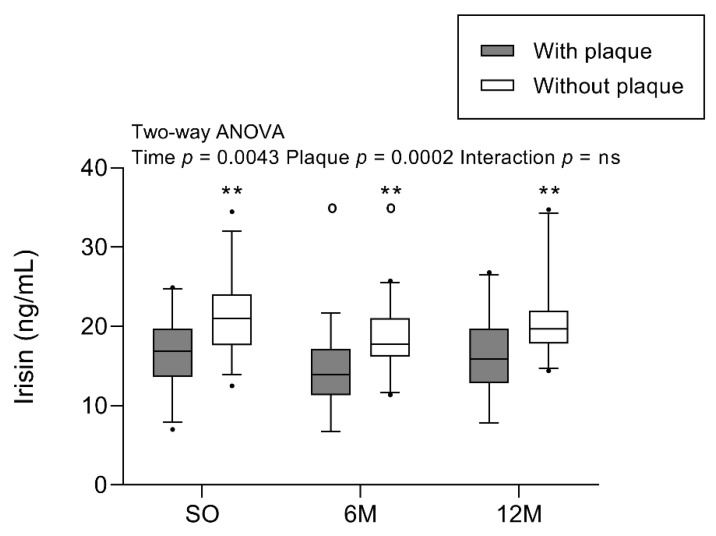
Irisin concentration in participants with SO with/without plaques before and after BS. Results are represented in a boxplot showing the median and first to the third quartiles, with whiskers showing the 5th and 95th percentiles. Points below and above the whiskers are drawn as individual points. Two-way ANOVA (group and time of follow-up) was performed with the Bonferroni post-hoc test. *n* = 61 subjects; *n* = 30 with plaque, and *n* = 31 without plaque. Statistical differences were considered significant when *p* < 0.05. Symbol (^o^) denotes differences in the post-test vs. SO for the same group. Symbol (*) denotes differences in the post-test vs. with-plaque group for the same time. One or two of either symbols denote *p* < 0.05 or *p* < 0.01, respectively. Abbreviations: 6M, 6 months after surgery; 12M, 12 months after surgery; BS, bariatric surgery; ns, non-significant; SO, severe obesity at 1 month before bariatric surgery.

Figure 1 shows irisin concentrations 1 month before and at 6 and 12 months after BS for the groups with and without plaques. When comparing the differences between participants with and without plaque, irisin concentrations were significantly lower in those with atheromatous plaques before and after BS (*p* = 0.0002). When focusing on the effect of BS, irisin concentration decreased 6 months post BS and increased again after one year (time *p* = 0.0043). Overall, irisin levels were significantly different during the study and depended on the presence of plaques.

**Figure 2:**
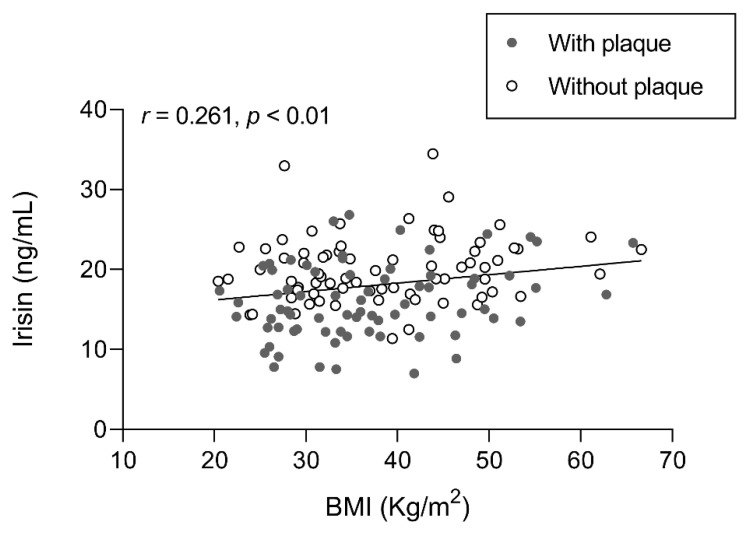
Association of irisin levels with BMI in participants with SO with/without atheromatous plaques before and after BS. Results were obtained after Pearson’s test and expressed as the Pearson’s coefficient (*r*). *n* = 61 subjects. In gray, subjects with plaque; in white, subjects without plaque. Abbreviations: BMI, body mass index.

To delineate the association of irisin levels with obesity and weight loss, we studied the correlation between irisin levels and BMI at each follow-up (Figure 2). This revealed a positive correlation (*r* = 0.261; *p* < 0.01) with higher irisin levels in individuals with higher BMIs. The reported correlation can only be observed globally, not separately at each time point.

Next, we measured the distribution of *FNDC5* rs3480 polymorphism frequencies by plaque presence to assess whether the reported differences in irisin levels reflected genetic variability (Table 2). Nevertheless, there was no significant difference in the allele and genotype frequencies between individuals with and without atheromas.

Three-factor ANOVA was then used to determine if the genotype alone and combined with plaque or time after surgery affected irisin concentrations (Table 2). This revealed no statistically significant differences in the irisin levels by the genotype alone (*p* = 0.0952). Furthermore, the interactions of the genotype with plaque presence or time were not statistically significant (*p* = 0.8805 and *p* = 0.9113, respectively).

### 2.3. Association between Irisin Levels and Biochemical Features of Atherosclerosis

The parameters implicated in atherogenesis, such as inflammation, oxidative stress, and endothelial function, were previously measured in this cohort [9,19,20]. Table 3 shows the analysis of the correlations between irisin and some of these parameters. Of note, we found a negative correlation with pro-inflammatory parameters, such as non-esterified fatty acids (NEFA) and plasminogen activator inhibitor 1 (PAI1), and a positive correlation with leptin. The levels of oxidized low-density lipoprotein cholesterol (a marker of oxidative stress) correlated negatively with irisin levels. Endothelial function, including the inflammatory adhesion molecule P-selectin and the carotid intima-media thickness (cIMT), also correlated negatively with irisin.

### 2.4. Irisin as a Biomarker of Subclinical Atherosclerosis

**Figure 3:**
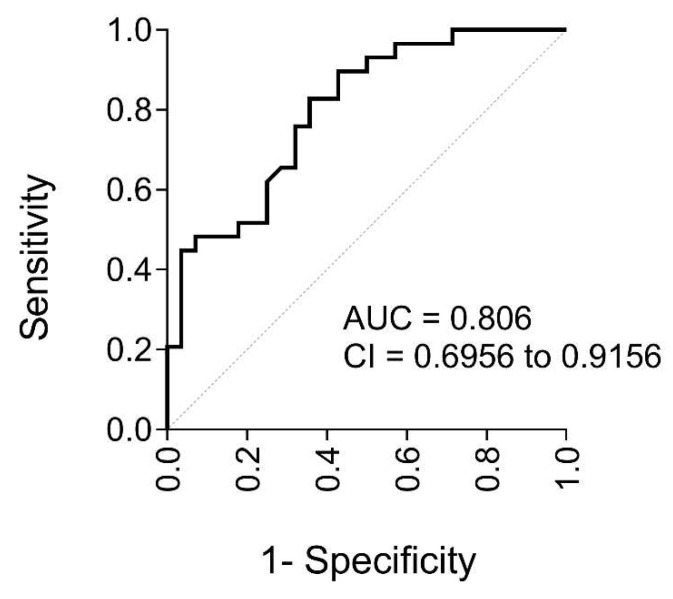
Goodness of irisin level prediction in participants with SO before BS. Quantified using ROC curve analysis with the AUC and its 95% CI (*n* = 61 subjects). The dotted line is the reference line (AUC = 0.5). Abbreviations: AUC, area under the ROC curve; CI, confidence interval; ROC, receiver operating characteristic.

Given the differences between the groups with and without atheromas, and the correlations of irisin with atherogenesis-related parameters, we evaluated the potential of irisin as a biomarker for subclinical atherosclerosis. The area under the receiver operating characteristic (ROC) curve (AUC) was used to calculate the accuracy of irisin as a potential diagnostic test (Figure 3). The high AUC (0.81; i.e., 81% predictive capacity) indicated that irisin levels have good overall accuracy for discriminating the presence of plaque.

## 3. Discussion

Identifying subjects with subclinical atherosclerosis is crucial to enhance the diagnosis and prevention of CVD. We found that irisin may not only be a good biomarker for subclinical atherosclerosis, but could also be related to the SO itself.

Studies on the potential relationship between irisin levels and BMI or obesity have shown inconsistent findings [21]. Our results were consistent with the most frequently reported finding that plasma irisin levels correlate positively with BMI [22]. It has been suggested that adipose tissue could oversecrete irisin to tackle obesity-induced metabolic dysregulation [23], thereby explaining the observed positive association between irisin and leptin in SO [9,24]. Leptin is an adipokine that reflects dysregulated metabolism.

When we studied circulating irisin levels after surgery, a marked reduction was observed at 6 months following BS. However, at 12 months, the levels had returned to their pre-surgical baseline. These findings are consistent with previous research indicating a decrease in *FNDC5* expression in muscle tissue after BS [25]. However, it remains to be confirmed whether the decrease in circulating irisin levels at 6 months were due to decreasing muscle mass, the surgery itself, or a direct effect of the resulting weight loss. Recovery to the initial values 12 months after BS has been suggested to reflect adaptation to the altered energy intake and body weight [26]. Extended investigations are required to determine whether irisin levels remain stable over the long term after BS.

The observed correlation between irisin and BMI suggests that irisin could also be a key molecule in atherosclerosis because most processes influenced by irisin are also disrupted in atherosclerosis. Indeed, low serum irisin levels have been linked to increased risk of CVD [27], and more recently, to the presence of plaques in patients with axial spondyloarthritis [28]. Despite these reports, little is known about the role of irisin in atherosclerosis and its exact mechanism of modulation. Hence, it is important to highlight that we found lower irisin levels in subjects with atheromas throughout the study, even after BS.

To complement the assessment of irisin at the protein level, we also assessed whether the observed differences resulted from genetic variability. Although previous research has reported contradictory findings on this association [29], irisin polymorphisms have been associated with obesity and related comorbidities [30]. The rs3480 noncoding polymorphism of *FNDC5* is of interest here, given its association with blood pressure and lipid profiles [31]. Furthermore, the G allele of rs3480 has been associated with increased risk of myocardial infarction [32]. However, our analysis of the *FNDC5* rs3480 polymorphism and its possible association with the presence of plaques revealed that is not associated with plaque presence and did not modulate irisin concentrations at any assessment point. Hence, plasma irisin levels were independent of any variability in the rs3480 polymorphism.

The positive correlation of irisin with BMI and the increase in irisin levels among patients without plaques support the hypothesis that irisin has an atheroprotective role [13,33,34,35]. Different explanations support this effect of irisin against the development of atherosclerosis in SO. These include indirect crosslinks that exist between irisin and comorbidities [36,37] and/or its role in the inflammation and oxidative stress processes leading to endothelial dysfunction [28], one of the first stages of atherosclerosis [38].

These results are further supported by our finding of a negative association between irisin levels and both glucose and HbA1c, which is indicative of the adverse blood sugar levels in our population before BS. Irisin appears to improve insulin resistance and T2DM [39], with evidence that increased levels in SO may be protective against T2DM, which is a known risk factor for CVD [40].

The anti-inflammatory and anti-oxidative potentials of irisin are of great interest because these processes are often associated with the development of atherosclerosis [41,42]. We have previously demonstrated that SO is often characterized by increased inflammation [9,19]. Supporting the anti-inflammatory function of irisin [41], we also reported that plasma irisin was negatively correlated with non-esterified fatty acid levels, which can induce inflammation by modulating adipokine production [43]. It is also worth highlighting that there exists an inverse relationship between irisin and plasminogen activator inhibitor-1 levels, a recognized risk factor for thrombosis and atherosclerosis in inflammatory conditions [44].

Consistent with other reports demonstrating that irisin reduces oxidative stress [45], it correlated negatively with the levels of oxidized low-density lipoprotein cholesterol, a well-known marker of oxidative stress that contributes to the formation and progression of atherosclerotic plaques [46].

Irisin may also help to prevent endothelial dysfunction and damage [15,41,47,48], which are crucial to the development of CVD. We previously demonstrated that our population with SO and early atherosclerosis had evidence of endothelial dysfunction [9]. Supporting the potential protective role of irisin, the present study indicates a strong negative correlation with P-selectin, an adhesion molecule that indicates the presence of inflammation in the vascular bed and enhances pro-coagulant activity [49]. Importantly, circulating irisin levels also correlated negatively with the carotid intima-media thickness, a characteristic morphological change in early atherosclerosis [50]. Accordingly, several studies have reported that irisin may suppress neointima formation, which reflects endothelial injury in the atherosclerotic vasculature [51,52]. Thus, lower irisin levels might reflect a worse pathological status in patients with early atherosclerosis.

Given the important functions of irisin and its beneficial effects on homeostasis [53], together with the many correlations highlighted in this research, we suggest that irisin could serve as a potential biomarker of subclinical atherosclerosis. AUC analysis of how well irisin distinguishes between subjects with and without plaques indicated an 81% predictive capability, which suggests that irisin alone can determine the presence of plaques. This fact, combined with the difficulty of diagnosing atherosclerosis by ultrasound in patients with SO, emphasizes the usefulness of irisin as a biomarker of subclinical atherosclerosis in this population. To the best of our knowledge, this is the first study to consider this possibility.

We are aware that this exploratory research has some limitations, such as a small sample size. It would also have benefited from additional data on body composition (muscle and fat mass) to gain a better understanding of the role of irisin in obesity metabolism. Measuring plaque composition could provide valuable information about irisin as a marker of atherosclerosis degree and severity. Further studies are needed to confirm or refute our hypothesis about the usefulness of irisin as a biochemical marker of atherosclerotic plaque presence. Nevertheless, the differences in irisin levels between subjects with and without atheroma are noteworthy and open up the possibility to use irisin as a biomarker of subclinical atherosclerosis.

## 4. Materials and Methods

### 4.1. Study Design and Participants

This prospective observational study included 61 participants with SO recruited at the Arnau de Vilanova University Hospital (Lleida, Spain) after being enrolled in out BS program, with follow-up at 6 and 12 months after BS. The study was conducted in accordance with the Declaration of Helsinki and was approved by the Ethics Committee of Hospital Universitari Arnau de Vilanova (CEIC-1369). For inclusion, participants were required to have a body mass index (BMI) of >40 kg/m^2^ or >35 kg/m^2^ with at least one of the following comorbidities: hypertension, T2DM, dyslipidemia, obstructive sleep apnea, and weight-induced rheumatic disease [54]. All presented a clinical history of SO for at least 5 years, none had received an inflammatory or infectious disease diagnosis, and none received anti-obesity or anti-inflammatory drugs during the study. We excluded cases with previous neoplastic, renal, hepatic, or active systemic diseases, as well as those with a CVD or endocrine disease other than T2DM.

At the study onset, 61 individuals were distributed into two groups based on the presence (*n* = 30) or absence (*n* = 31) of atherosclerotic plaques, which we detected using a Vivid I ultrasound machine (General Electric Healthcare, Waukesha, WI, USA) [55]. Arterial ultrasound was performed in the common, bifurcation, internal, and external regions of both carotid arteries [55]. Participants underwent either laparoscopic Roux-en-Y gastric bypass (*n* = 31) or sleeve gastrectomy (*n* = 30) at the surgeon’s discretion. Plasma samples were obtained 1 month before and at 6 and 12 months after BS. Subcutaneous adipose tissue samples were obtained during BS for genotyping assays.

### 4.2. Clinical and Biochemical Evaluations

Serum levels of total cholesterol, low-density lipoprotein cholesterol, high-density lipoprotein cholesterol, and triacylglycerides were measured using colorimetric enzymatic methods (CHOD-PAP cholesterol, HDL-C plus, and GPO-PAP triglycerides, Modular P800; Roche Diagnostics, Manheim, Germany). Glucose was determined by the hexokinase glucose method; HbA1c was measured using a high-resolution ion exchange liquid chromatography technique (Varian II Hemoglobin A1c; Bio Rad, Hercules, CA, USA) and HOMA-IR was then calculated [56]. Plasma irisin concentrations were measured by enzyme-linked immunosorbent assay ELISA (Ref: EK-067-29; Phoenix Europe GmbH, Karlsruhe, Germany).

### 4.3. Irisin Polymorphism Selection and Genotyping

All participants underwent genotyping for the irisin rs3480 (A/G) polymorphism, which has previously been linked to CVD. We extracted DNA from subcutaneous adipose tissue samples using the Canvax Extraction kit (Tissue Genomic DNA Purification Kit; Canvax Biotech SL, Córdoba, Spain). The concentration of the extracted DNA was measured by the fluorometer Qubit with two standards (High Sensitivity Qubit Kit; Life Technologies; ThermoFisher Scientifics, Waltham, MA, USA). The rs3480 single nucleotide polymorphism was genotyped using the TaqMan 5′ exonuclease assay (Applied Biosystems; Foster City, CA, USA). Assays were run in a 384-well plate on QuantStudio 6 and 7 Pro Real-Time PCR Systems, using standard conditions. Genotype data were analyzed using the Design and Analysis Software (version 2.5, Applied Biosystems, Foster City, CA, USA).

### 4.4. Statistical Analysis

Data were analyzed in GraphPad Prism (version 8.1, GraphPad Software, San Diego, CA, USA) with an alpha level of 0.05 used to determine statistical significance. The Shapiro–Wilk test was used to determine the normality of continuous variables, which were reported as means ± standard error of the mean. By contrast, categorical variables were represented as percentages (yes/no). Box plots were used to indicate the medians and first to third quartiles of the distribution.

Statistical analysis of age and sex was performed using the Mann–Whitney *U* test. For categorical variables, chi-squared or Fisher exact tests were performed. For continuous variables, two-way ANOVA with the Bonferroni post-hoc test was used to evaluate differences between groups. Differences in genotype frequencies were assessed by the chi-squared test, while the determination of whether the genotype affected irisin concentrations or plaque presence was determined by time three-way ANOVA. The correlation of biomolecules with irisin was assessed by Pearson’s test. Finally, ROC curve analysis was used to describe the discriminatory accuracy of irisin for subclinical atherosclerosis when used as the primary method to determine the presence of disease as a dichotomous outcome.

## 5. Conclusions

Plasma irisin concentrations were positively associated with BMI in patients with SO undergoing BS. Irisin overexpression might act by tackling the deregulated metabolism induced by obesity and exerting a protective role against atherosclerosis. Overall, irisin appears to have a high predictive value for the presence of atherosclerotic plaques, and either alone or in combination with other markers, it could offer a valuable tool for the diagnosis of subclinical atherosclerosis in SO. This might improve the accuracy and detection of plaques at earlier stages, thereby helping to prevent acute CVD.

## Figures and Tables

**Table 1:** Clinical characteristics of participants with SO with/without plaques before and after BS. Continuous variables are expressed as the mean ± standard error of the mean. Statistical analysis for age and sex used Mann–Whitney *U* tests. Continuous variables were analyzed by two-way ANOVA (group and time of follow-up) and the Bonferroni post-hoc test. Statistical analyses for categorical variables were performed using chi-square or Fisher exact tests. Symbol (^o^) denotes differences vs. SO; symbol (*) denotes differences vs. with-plaque group. One, two, or three of these symbols denotes *p* < 0.05, <0.01, or <0.001, respectively. Abbreviations: 6M, 6 months after surgery; 12M, 12 months after surgery; BMI, body mass index; BS, bariatric surgery; cHDL, high-density lipoprotein cholesterol; cLDL, low-density lipoprotein cholesterol; DLP, dyslipidemia; HbA1c, glycated hemoglobin; HOMA-IR, Homeostatic Model Assessment for Insulin Resistance; HT, hypertension; *n*, number of subjects; ns, non-significant; SO, severe obesity at 1 month before bariatric surgery; T2DM, type 2 diabetes mellitus; TAG, triacylglyceride; TC, total cholesterol.

Variables		With Plaque (*n* = 30)			Without Plaque (*n* = 31)			*p* Value
		SO	6M	12M	SO	6M	12M	Plaque/Time/Interaction
Anthropometrical	Sex (women/men)	16/14	-	-	26/5	-	-	0.0134
	Age (years)	51.9 ± 1.9	-	-	43.2 ± 1.8	-	-	0.0021
	BMI (kg/m^2^)	46.5 ± 1.3	32.0 ± 1.0 ^ooo^	29.5 ± 0.8 ^ooo^	48.8± 1.2	33.0 ± 0.9 ^ooo^	29.7 ± 0.8 ^ooo^	ns/<0.0001/ns
Biochemical	TC (mg/dL)	185.0 ± 8.4	161.5 ± 8.6 ^o^	181.4 ± 7.5	173.9 ± 4.8	150.7 ± 5.7 ^oo^	163.0 ± 7.4	ns/<0.0001/ns
	cHDL (mg/dL)	42.9 ± 1.7	44.5 ± 1.7	53.2 ± 1.9 ^ooo^	46.2 ± 1.4	45.3 ± 1.5	55.2 ± 2.1 ^ooo^	ns/<0.0001/ns
	cLDL (mg/dL)	109.1 ± 7.1	91.5 ± 6.5 ^o^	108.1 ± 6.7	104.5 ± 4.7	86.7 ± 5.3 ^o^	93.8 ± 7.2	ns/0.0024/ns
	TAG (mg/dL)	162.4 ± 14.8	116.9 ± 10.3 ^ooo^	98.4 ± 11.5 ^ooo^	120.4 ± 7.2 **	92.2 ± 4.5 ^o^	74.9 ± 4.6 ^ooo^	0.0073/<0.0001/ns
	Glucose (mg/dL)	125.4 ± 7.2	90.9 ± 3.6 ^ooo^	100.6 ± 12.9 ^o^	97.2 ± 3.1 **	81.1 ± 1.7	82.7 ± 1.8	0.0028/<0.0001/ns
	HOMA-IR	5.2 ± 0.9	1.2 ± 0.4 ^o^	1.3 ± 0.4 ^o^	1.8 ± 0.4 *	0.7 ± 0.1	0.5 ± 0.1 ^o^	0.0029/0.0001/0.0091
	Hba1C (%)	6.61 ± 0.25	5.57 ± 0.22 ^ooo^	5.54 ± 0.15 ^ooo^	5.70 ± 0.16 ***	5.16 ± 0.11 ^o^	5.13 ± 0.07 ^o^	0.0060/<0.0001/ns
Comorbidities	Healthy (no HT, DLP and DM2) yes/no (%)	10/90	50/50 ^oo^	60/40 ^ooo^	55/45 ***	68/32	81/19 ^o^	
	HT yes/no (%)	77/23	43/57 ^oo^	33/67 ^ooo^	35/65 ***	26/74 *	13/87 ***^,o^	-
	DLP yes/no (%)	70/30	33/67 ^ooo^	17/83 ^ooo^	26/74 ***	13/87 **^,o^	6/94 *^,oo^	-
	T2DM yes/no (%)	67/33	30/70 ^ooo^	20/80 ^ooo^	16/84 ***	6/94 *	0/100 *^,o^	-

**Table 2:** Genotype frequencies of the *FNDC5* gene rs3480 (A/G) polymorphism and irisin levels in participants with SO with/without plaques. Genotype frequencies are presented as number of subjects and the percentage versus the total per group. Irisin concentrations are represented as the mean ± standard error of the mean. Abbreviations: SO, severe obesity at 1 month before bariatric surgery.

	With Plaque (*n* = 30)				Without Plaque (*n* = 31)			
rs3480 Genotype (A/G)	Irisin (ng/mL) (Mean ± SEM)				Irisin (ng/mL) (Mean ± SEM)			
	Frequencies *n* (%)	SO	6M	12M	Frequencies *n* (%)	SO	6M	12M
AA	12 (40%)	15.4 ± 1.1	14.0 ± 1.8	14.3 ± 1.2	11 (35.5%)	20.9 ± 1.0	17.8 ± 1.0	19.8 ± 1.0
AG	11 (36.7%)	19.5 ± 1.6	15.8 ± 2.3	17.5 ± 1.9	14 (45.2%)	23.0 ± 1.5	19.3 ± 1.4	21.8 ± 2.2
GG	7 (23.3%)	14.1 ± 1.1	12.6 ± 1.1	16.9 ± 1.3	6 (19.4%)	19.2 ± 2.1	17.8 ± 1.7	20.3 ± 0.9

**Table 3:** Association of irisin levels with atherosclerosis features in participants with SO with/without plaques. Calculated in the total cohort with SO with/without plaques (before bariatric surgery or throughout the study). Statistical analyses were performed using Pearson’s test and expressed as the Pearson’s coefficient (*r*). *n* = 61 subjects. Abbreviations: cIMT, carotid intima-media thickness; HbA1c, glycated hemoglobin; NEFA, non-esterified fatty acids; oxLDL, oxidized low-density lipoproteins; PAI-1, plasminogen activator inhibitor-1; P-sel, P-selectin; SO, severe obesity at 1 month before bariatric surgery; T2DM, type 2 diabetes mellitus.

		Irisin (ng/mL)	
Process	Parameters	*r*	*p*
**Correlation before BS**			
**Inflammation**	**NEFA (mmol/L)**	−0.310	0.019
	**PAI-1 (ng/mL)**	−0.121	0.038
**Correlation all the study times**			
**Metabolism**	**Leptin (ng/mL)**	0.263	0.002
**T2DM**	**Glucose (mg/dL)**	−0.359	0.006
	**Hba1c (%)**	−0.421	0.003
**Oxidative stress**	**oxLDL (U/L)**	−0.226	0.006
**Endothelial function**	**P-sel (ng/mL)**	−0.399	<0.001
	**cIMT (mm)**	−0.182	0.025

## Data Availability

Data are unavailable due to privacy or ethical restrictions.
